# Negative regulation of REST on NR2B in spinal cord contributes to the development of bone cancer pain in mice

**DOI:** 10.18632/oncotarget.9447

**Published:** 2016-07-30

**Authors:** Dan Wang, Jianbo Yu

**Affiliations:** ^1^ Department of Anesthesiology, Tianjin Nankai Hospital, Tianjin Medical University, Tianjin, China

**Keywords:** bone neoplasms, pain, REST, NR2B protein, mice

## Abstract

In this study, C3H/HeNCrlVr mice are implanted with sarcoma NCTC 2472 cells into the intramedullary space of the femur to induce ongoing bone cancer-related pain behaviors. During the progress of the bone cancer pain, the down-regulation in spinal REST (Neuron-restrictive silencer factor, NRSF/REST) with concomitant up-regulation in spinal NR2B (2B subunit of N-methyl-D-aspartate receptor, NR2B) protein expression are observed at days 5, 7, 10 and 14 post-inoculation. Immunofluorescence assay shows that almost all of REST and NR2B-positive signals encompass NeuN (neuron-specific nuclear protein, a neuronal marker)-positive signals in spinal cord of sham and tumor-bearing mice. Different from previous researches involved in the main distribution of REST in neural progenitors, the expression of REST in mature neurons in spinal cord of adult mice is observed. Intrathecal administration of AS-ODN of REST at days 0, 2, 4 and 6 post-inoculation further enhances expression of spinal NR2B at day 7 post-inoculation, which suggests the reduced suppression of spinal REST on NR2B during the development of bone cancer pain. In summary, our study provides the evidence that the negative regulation of REST on NR2B in spinal cord takes part in the exacerbation of bone cancer pain.

## INTRODUCTION

It is well established that both peripheral and central hypersensitivity participate during the progress in bone cancer pain. Though researches on peripheral mechanism of the bone cancer pain go further, the central mechanism is considerably complex and not well understood. The neural synaptic plasticity, as known as the ability of the nervous system to alter adapted to external events, is critical for central sensitization [1]. NMDA receptor (NMDAR) activation has been proved as a key role for the production and maintenance of central sensitization [2]. Advances reveal that changes in NMDAR-binding synaptic plasticity induce long-term potentiation [3, 4] and generate epigenetic spinal hypersensitivity [5-9]. Our previous studies indicated that spinal NR2B was present at a critical locus involved in exacerbation of bone cancer pain, which was observed with long-lasting up-regulation with the progressive of pain behavior [10-13]. Ifenprodil, the selective antagonist of NR2B, attenuated tumor-induced pain behavior in a dose-dependent manner [10, 14]. This study investigates the upstream mechanism of NR2B to observe the immediate modulation on NR2B in bone cancer pain in spinal cord.

REST is a transcriptional repressor which widely expresses in pluripotent stem cells and neural progenitors, where it acts as a negative modulator *via* epigenetic remodeling to silence coding or non-coding neuronal genes which are important for neurogenesis and synaptic fuction [15-21, 31]. REST specifically combines with dsDNA of neuron-restrictive silencer element (NRSE, also known as RE1), a 23-base-pair motif that is highly conserved in transcriptional regulation regions of neuron-specific genes, which repressed the transcription of downstream gene of RE1 *via* histone deacetylation [22-25, 31]. Previous research revealed that there were five highly conserved putative RE1 binding sites which, located in the 5’-flanking region of NR2B promoter, repressed the transcription of Grin2b *via* recruiting REST [18]. The present study investigates whether the mechanism of spinal REST-NR2B exists in the progress of bone cancer pain in mice as well as their cellular localization.

## RESULTS

### Pain behavior tests

**Figure 1:**
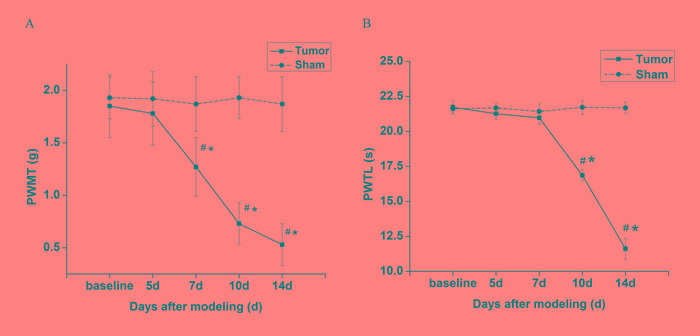
Changes of pain behaviors of the right hind limb over time in tumor-bearing mice and sham mice **A.** PWMT to von Frey filaments of tumor-bearing mice decreased over time after day 7. **B.** PWTL of tumor-bearing mice decresaed gradually after day 10. 5, 7, 10 and 14 d indicate days after innoculation. Data are expressed as means ± SD. ^#^*p* < 0.05 *vs* baseline, *p < 0.05 *vs* sham mice.

Compared with the baseline value and sham group, the right hind limb of tumor mice displayed a statistic decrease of PWMT at day 7 post-implantation (Figure 1A), while a statistic decrease of PWTL was observed at day 10 post-implantation (Figure 1B). Pain behaviors of tumor mice were aggravated as time gone by, especially on day 14 (*p* < 0.05). Nevertheless, no significant decrease in PWMT and PWTL was observed in sham mice at all time points.

### Expression changes of spinal NR2B and REST protein during bone cancer pain before intrathecal injection

**Figure 2:**
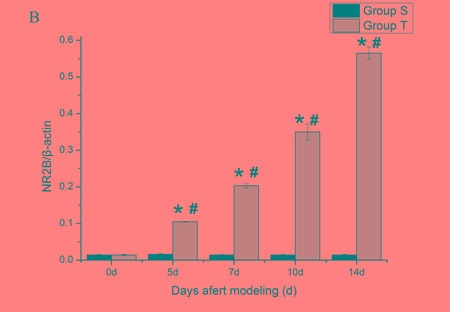
Mice spinal NR2B protein changes during the bone cancer pain The expression of spinal NR2B protein increased with the development of tumor-evoked pain behavior. **A.** Bands of western blotting of the NR2B protein expression (200 KDa). β-actin is a loading control. **B.** Statistical analysis of relative density of western blotting between sham and tumor mice. 5 d, 7 d, 10 d and 14 d indicate days after inoculation. *p < 0.05 *vs* the same time point of sham mice, ^#^*p* < 0.05 *vs* the same mice on day 0.

**Figure 3:**
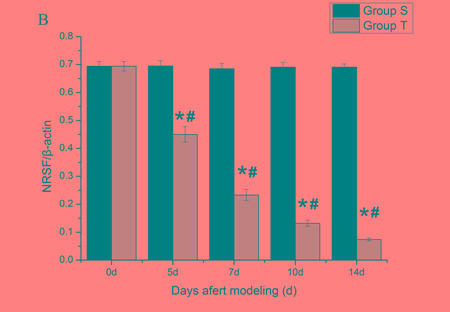
Mice spinal REST protein changes during the bone cancer pain The expression of spinal REST protein decreased with the development of tumor-evoked pain behavior. **A.** Bands of western blotting of the REST protein expression (116 KDa). β-actin is a loading control. **B.** Statistical analysis of relative density of western blotting between sham and tumor mice. 5 d, 7 d, 10 d and 14 d indicate days after inoculation. *p < 0.05 *vs* the same time point of sham mice, ^#^*p* < 0.05 *vs* the same mice on day 0.

To quantify the expression level of NR2B and REST protein, western blot analyses were performed. Compared with normal mice (day 0) and sham mice, the level of spinal NR2B in tumor mice was enhanced gradually at all post-inoculation tests. However, the level of REST decreased at day 5 post-inoculation and reduced progressively with the increasing bone pain. There was no significant different among sham group in overall time points (Figure 2 and Figure 3).

### Expression change of spinal NR2B after 1 day intrathecal injection of AS-ODN

**Figure 4:**
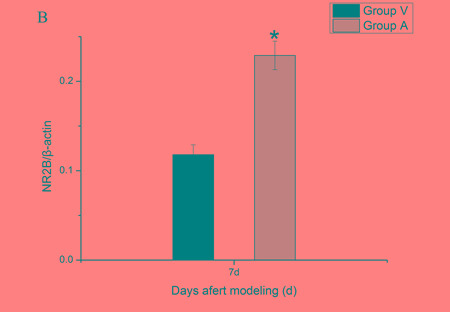
Spinal NR2B protein changes after intrathecal adminstration with AS-ODN AS-ODN increased the expression of the NR2B protein in mice spinal cord at day 7 after inoculation. **A.** Bands of western blotting of the NR2B protein expression (200 KDa). β-actin is a loading control. **B.** Statistical analysis of relative density of western blotting between group Vehicle and group AS-ODN mice at day 7 after inoculation. *p < 0.05 *vs* group Vehicle.

To investigate the putative role of spinal REST on NR2B in bone cancer pain, we examined the level of NR2B protein at 24 h after exogenous intervention (Figure 4). Compared with the tumor mice injected with vehicle (aCSF) by intrathecal route, AS-ODN did enhanced the level of spinal NR2B protein (*p* < 0.05).

### Bone destruction test

**Figure 5:**
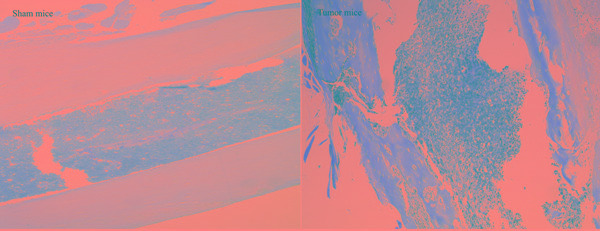
Quantification of bone destruction after injection of sarcoma cells (tumor mice) or culture medium (sham mice) into the femoral intramedullary space Hematoxylin-eosin staining of 14 days sham and 14 days tumor-bearing femur, showed the replacement of the lightly stained marrow cells with the more darkly stained sarcoma cells (original magnification×10). Radiographs of 14 days sham and 14 days tumor-bearing femur, showed the loss of bone caused by tumor growth (arrow).

To investigate the bone destruction induced by sarcoma, Hematoxylin-eosin and Radiographs were applied. Lots of sarcoma cells with abnormal forms and staining of nucleus were observed in tumor-bearing femoral medullary cavity on day 14 after inoculation. Compared with sham mice, X-ray of the ipsilateral femur displayed an obvious loss on day 14 (Figure 5).

### Cellular localization of REST and NR2B

**Figure 6:**
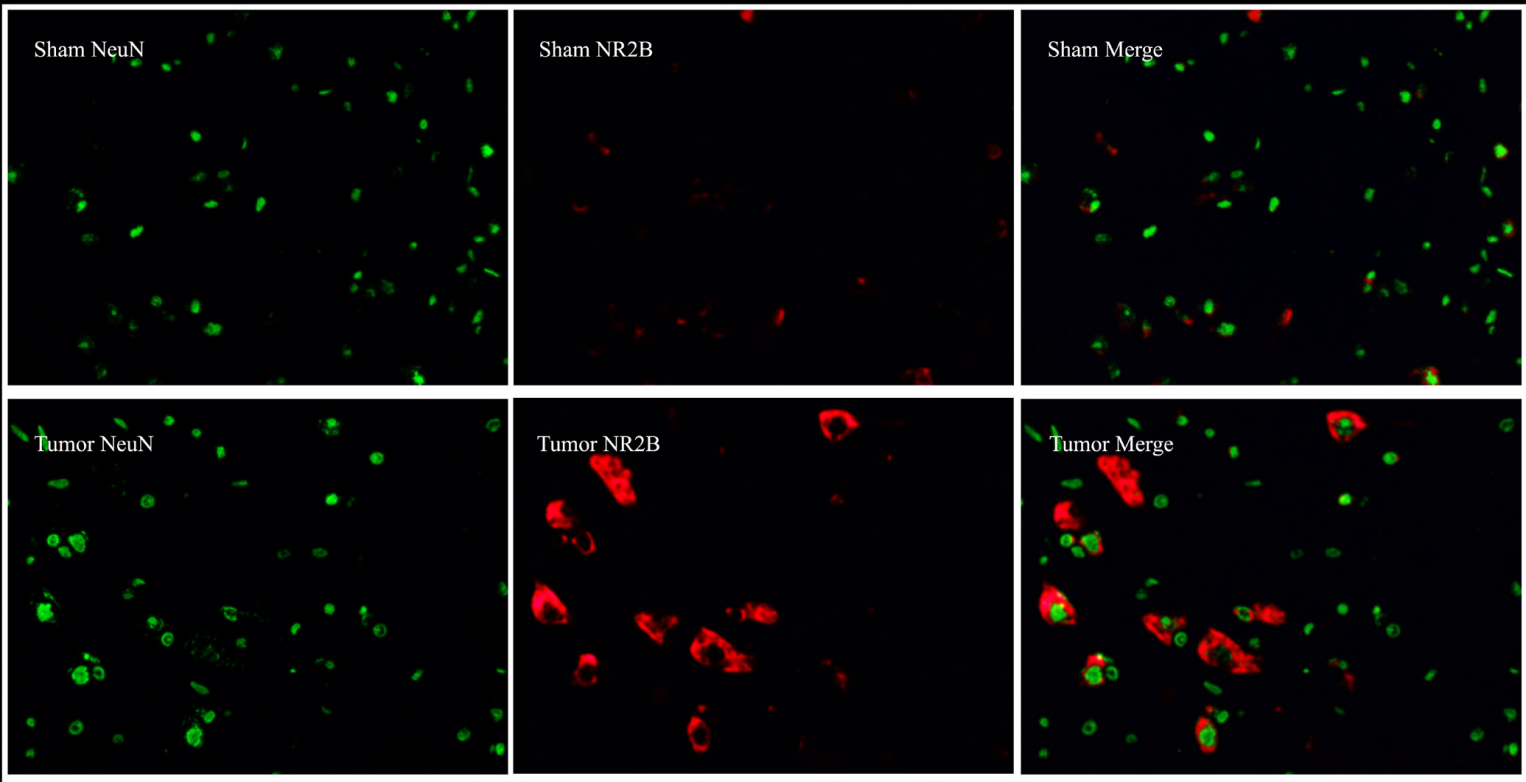
Immunofluorescence assays of localization of NR2B and REST protein in neurons using fluorescence microscope (original magnification×400) Immunohistochemical double labeling between REST (red) and NeuN (green), NR2B (red) and NeuN (green), in the lumbar enlargements of sham and tumor mice. Almost all of red signals encompassed green signals in neurons. Compared with sham group, the REST-positive signals showed an obvious decrease with concomitant increase of NR2B-positive signals.

**Figure 7:**
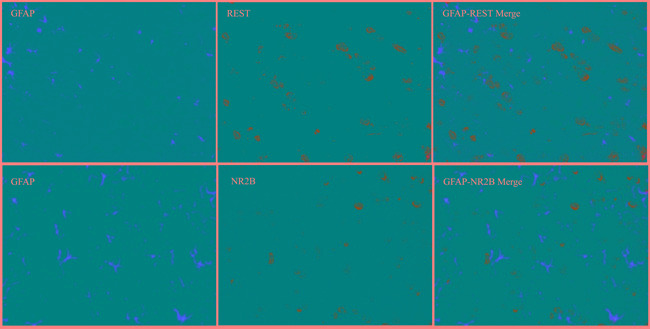
Immunofluorescence assays of the localization of NR2B and REST protein in astrocytes using fluorescence microscope (original magnification**×**400) Immunohistochemical double labeling between REST (green) and GFAP (red), NR2B (green) and GFAP (red), in the lumbar enlargements of tumor mice. Both green signals and red signals existed independently.

Immunofluorescence technique was used to observe the intracellular distribution of REST and NR2B in neurons or astrocytes. Almost all REST or NR2B-positive signals are colocalized with NeuN-positive signals in the lumbar enlargement of sham mice. Moreover, bone neoplasms markedly decreased REST-positive signals and increased NR2B-positive signals in NeuN-positive neurons at day 14 after inoculation (Figure 6). However, the REST, NR2B and GFAP (glial fibrillary acidic protein, the marker of astrocytes)-positive signals existed separately (Figure 7).

## DISCUSSION

In this experiment, we model *via* injecting the sarcoma cells in right femurs of male C3H/HeNCrlVr mice. Similar to the study reported by Schwei et al. and our previous researches [10, 13, 27], inoculation of sarcoma cells induces pain-related behavioral signs of mechanical allodynia and thermal hyperalgesia, and severe bone destruction during 14 days post-operation. The characteristic changes in X-ray and hematoxylineosin staining further manifests that the model is made successfully.

Here we demonstrate an important and previously unrecognized role for REST during the progress in bone cancer pain. Previous researches stated the expression of REST was declined to inappreciable level in the course of the differentiation of embryonic cells into mature neurons [15, 16]. However, in our research, the expression of REST is detected in the mature neurons in spinal cord of adult mice, which reveals that REST is also distributed in mature nervous system instead of only in embryo nervous tissues. This finding indicates that REST participates not only in the differentiation of neural progenitors but also in functional control of mature neurons. In addition, the decreasing expression of spinal REST with concomitant aggravating tumor-evoked pain behavior suggests to some extent the potential correlation between spinal REST and bone cancer pain.

Previous research indicated that REST was presented at a critical locus involved in the developmental switch in GluN2A/GluN2B ratio. REST down-regulated the expression of NR2B *via* orchestrating epigenetic remodeling of Grin2b promoter and repressing the long-lasting transcription of Grin2b [31]. This suppression of REST on NR2B might account for the opposite trend of the expression of spinal REST and NR2B in tumor-bearing mice. Intrathecal administration of AS-ODN of REST produces significant increase on expression of NR2B at 7 day after inoculation compared with the vehicle group, which reconfirms the reduced expression inhibition of spinal REST on NR2B during the progress of bone cancer pain. Previous study indicated that REST was combined with RE1 to repress the transcription *via* recruiting HDAC which induced hypoacetylation of histones [16, 32]. The long-lasting up-regulation of REST and RE1 complex in turn caused the continuous down-regulation of Grin2b [17, 33]. It has also been demonstrated that the 5’-flanking region of Grin2b contains five RE1-like elements which located between base pair -1407 and -2741 [18]. Given the researches above-mentioned, we propose that the persistent increase of NR2B expression along with the aggravation of bone cancer pain is stimulated at least in part by REST-binding transcription activation, though there is no in-depth addressing in terms of molecular mechanisms underlying the inverse regulation in present research.

*Via* immunohistochemical analysis, we find that both REST and NR2B exist in the cytoplasm of neurons instead of astrocytes. Furthermore, the change trend of positive signals of REST and NR2B show similar results to Western blot. These findings strongly suggest that the mechanism of REST-NR2B mainly exists in neurons but not in astrocytes.

In conclusion, our study demonstrates that osteocarcinoma induces tumor-evoked pain behaviors accompanied with the down-regulated expression of spinal REST and up-regulated expression of spinal NR2B. We find that spinal injection of AS-ODN of REST increases the expression of NR2B. The present research provides a basis for a latent role of the mechanism of spinal REST-NR2B during the progress of bone cancer pain. In-depth molecular mechanisms of REST-NR2B during the bone cancer pain need further investigation.

## MATERIALS AND METHODS

### Animals

The present experiments were approved by the Animal Care and Use Committee at Tianjin Medical University (Tianjin, China) and abided by guidelines for the use of laboratory animals [26]. Male C3H/HeNCrlVr mice (4-6 weeks old, Beijing Vital River Laboratory Animal Technology Co., Ltd., Beijing, China) weighing 20-25 g were used in all of experiments. Mice were housed 5 per cage in a temperature-controlled (21 ± 1 ºC) room with 12 h alternating dark/light cycles and fed with food and water ad libitum.

### Cell culture and implantation

NCTC 2472 sarcoma cells (American Type Culture Collection, ATCC) were cultured with NCTC 135 medium (Sigma-Aldrich, St. Louis, USA) (pH 7.4) containing 10% horse serum (Gibco, Grand Island, USA) under 5% CO_2_ at 37 ºC and passaged twice a week in accordance with ATCC recommendations. Implantation of sarcoma cells was performed as previously described by Schwei et al. [27] and Gu X et al. [10, 13]. Mice were anesthetized with intraperitoneal injection of 50 mg/kg pentobarbital sodium (2% in normal saline). Gonarthrotomy was performed to expose the femur condyle. A dental bur and a 30-gauge needle were used respectively to make a light depression and perforate the bone cortex, then a volume of 20 μl α-MEM containing no or 10^5^ NCTC 2472 cells was injected into the intramedullary space of the femur bone with a 25 μl microsyringe to make sham or tumor-bearing mice models. Afterwards, dental amalgam was used to seal the injection hole. The wound was closed.

### Oligonucleotide treatments

The antisense oligodeoxynucleotide (AS-ODN, 5’-CGGAAGGGCTT- GGCC-3’) was designed to target the mouse REST sequence. Artificial CSF (aCSF) was the solvent of the AS-ODN and was compounded with the following chemical composition (in mM): 3.8 KCl, 125 NaCl, 2.0 CaCl_2_, 1.0 MgCl_2_, 1.2 KH_2_PO_4_, 26 NaHCO_3_ and 10 glucose (pH 7.4). AS-ODN was intrathecally injected at a dose of 10 ug per 5 ul of aCSF on day 0 before operation and days 2, 4 and 6 after operation. Drugs were administered to conscious animals and were injected into the subarachnoid space through the intervertebral foramen at the 5th or 6th lumbar vertebra as previously described by Hylden and Wilcox [28].

### Pain behaviors testing

Mice were randomly divided into tumor group (Group T) and sham group (Group S). Pain-related behaviors were tested at day 0 before operation and days 5, 7, 10 and 14 after operation. All trials were performed during the light phase. Mice were allowed to have at least 30 min for habituation before each trial. All pain-related behaviors were detected by one laboratory technician who was blind to the treatment groups. Mechanical allodynia was assessed using von Frey ﬁlaments (Stoelting, Wood Dale, IL, USA). The method described by Chaplan et al. [29] was improved according to our previous researches [10, 13] for pain evaluation in mice. Mice were placed on a wire mesh platform (graticule: 0.5 cm×0.5 cm) with individual plexiglass compartments (10 cm×10 cm×15 cm). A set of von Frey ﬁlaments with the bending force ranged from 0.16 g to 0.20 g (0.16, 0.4, 0.6, 1.0, 1.4 and 2.0 g) were used to measured the paw withdrawal mechanical threshold (PWMT) by up-and-down method. The von Frey ﬁlaments were poked uprightly against the plantar surface for 6 to 8 seconds with an inter-stimuli interval of approximately 15 seconds. Vigorous paw withdrawal or paw ﬂinching was counted as positive responses. Each mouse was tested 5 times per stimulus strength and the lowest von Frey ﬁlaments which had at least 3 positive responses were regarded as PWMT. The paw withdrawal thermal latency (PWTL) to radiant heat according to the method described by Hargreaves et al. [30] and our previous studies [10, 13] was applied to assess the thermal hyperalgesia. Mice were placed on a 3 mm-thick-glass floor, covered with individual transparent plexiglass compartments (10 cm×10 cm×5 cm). A radiant thermal stimulator (BME410AInstitute of Biological Medicine, Academy of Medical Science, China) was focused onto the plantar surface of the hind paw through the glass floor. The characteristic lifting or licking of the hind paw was considered as the nociceptive end-points in the test, and the time to the end-points was regarded as PWTL. A cut-off time of 25 seconds was set up to prevent tissue injury. Each mouse was tested 5 times. There was an inter-stimuli interval of 5 minutes between trials. PWTL was the mean value of three mid-values.

### Western blot

While under deep anesthesia (5% sevoflurane), the L4-L5 segments of spinal cord were isolated quickly and stored in -80 ºC. Tissue samples were homogenized in lysis buffer and centrifuged at 13,000 rpm for 10 min at 4 °C. Supernatant was removed. Bradford method was used to determine the protein concentration. Samples (70 μg) were separated on SDS-PAGE (6%) and transferred onto a nitrocellulose membrane. The filter membranes were blocked with 5% nonfat milk for 1 h at RT (room temperature) and incubated with the primary anti NR2B (rabbit affinity purified polyclonal antibody; 1:1000, Abcam, Hong Kong, China), anti REST (rabbit affinity purified polyclonal antibody; 1:300, Santa Cruz Biotechnology, Santa Cruz, CA) or anti β-actin (mouse affinity purified monoclonal antibody; 1:1000, ZSGB-BIO, Beijing, China). The membrane was washed with TBST buffer and incubated for 1h with the secondary antibody conjugated with horseradish peroxidase (1:5000) for 1 h at RT and visualized in ECL solution followed by film exposure. The density of specific bands was quantified with a computer-assisted imaging analysis system (IPLab software, Scanalytics, Fairfax, VA).

### Immunofluorescence assay

Paraffin-embedded tissue section (5 μm) were dewaxed in xylene and rehydrated in graded ethnol solutions. The antigen retrieval method with citric acid solution was performed for 3 min at 100 °C, and then was washed in PBS. Following that, the tissues were permeabilized with 0.5% Triton X-100 for 15 min and blocked in normal goat serum at RT for 20 min. The sections were incubated with blocking buffer containing 3% BSA in PBST and subsequently reacted with anti REST (rabbit affinity purified polyclonal antibody; 1:100, Santa Cruz Biotechnology, Santa Cruz, CA) or anti NR2B (rabbit affinity purified polyclonal antibody; 1:200, Abcam, Hong Kong, China) overnight at 4°C. After washing, the sections were incubated with secondary antibody, Rhodamine or Fluorescein-conjugated AffiniPure goat anti-rabbit IgG (1:100, ZSGB-BIO, Beijing, China), for 20 min at 37 °C. For double immunolabeling, we used the following antibodies: mouse monoclonal antibody against NeuN (1:500; Abcam, Hong Kong, China), mouse monoclonal antibody against GFAP (1:200, Abcam, Hong Kong, China) and Fluorescein or Rhodamine-conjugated AffiniPure goat anti-mouse IgG (1:100; ZSGB-BIO, Beijing, China). After washing, the sections were mounted with PermaFluor and analyzed using a fluorescence inverted microscope (Olympus IX71, Tokyo, Japan).

### Statistical analysis

All data were expressed as mean ± SD. Data analysis was performed with the use of SPSS 16.0 software package for Windows. Animals were assigned to different groups in a randomized way. Repeated-measures ANOVA was performed to determine overall differences at each time point in PWMT and PWTL. ANOVA was used to measure the differences in the expression of NR2B or NRSF across all experiment groups and post hoc analysis was performed using the LSD test. A *P* value < 0.05 was deemed statistically signiﬁcant.
